# Determinants of sexual dysfunction in pregnancy in a large tertiary hospital in Ghana

**DOI:** 10.1371/journal.pone.0288456

**Published:** 2023-07-20

**Authors:** Ernest Tei Maya, Martin Owusu Boamah, Kofi Agyabeng, Emmanuel Srofenyoh, Kareem Mumuni, Ali Samba

**Affiliations:** 1 School of Public Health, University of Ghana, Accra, Ghana; 2 Department of Obstetrics and Gynecology, Greater Accra Regional Hospital, Accra, Ghana; 3 Department of Obstetrics and Gynaecology, University of Ghana Medical School, Accra, Ghana; 4 Department of Obstetrics and Gynaecology, Korle-bu Teaching Hospital, Accra, Ghana; Usmanu Danfodiyo University, NIGERIA

## Abstract

**Objective:**

To determine the factors associated with sexual dysfunction in pregnancy.

**Methods:**

A cross-sectional facility-based study using quantitative methods was carried out among pregnant women attending antenatal clinic of the Greater Accra Regional Hospital from 14^th^ May to 25^th^ June 2018. Four hundred and twenty-seven married or cohabiting women who were at least eight weeks pregnant and have been living together with their partners for at least four weeks prior to the survey were consecutively recruited. The Female Sexual Function Index (FSFI) tool was used to assess their sexual function. Pearson’s Chi Square, Fischer’s exact, Mann Whitney and Student’s t-tests were used for bivariate analysis where appropriate between sexual dysfunction (dependent variable) and demographic, obstetrics and gynecologic factors (independent variables). Multiple logistic regression was done. Statistical significance was set at p-value of less than 0.05 at bivariate and multivariable analyses.

**Results:**

The mean age of the respondents was 30.8 ± 4.8 years. Their mean gestational age was 32.3 ± 7.1 weeks. Marital status and duration of stay in marriage or cohabitation were significantly associated with sexual dysfunction with adjusted odds ratios of 1.88 (p-value < 0.05) and 1.08 (p-value < 0.05) respectively.

**Conclusion:**

Cohabiting and increasing length of stay with spouse are significantly associated with sexual dysfunction in pregnancy.

## Introduction

Sexual health is a fundamental human right according to the World Health Organization [1]. However, issues of female sexual function (FSD) have received incommensurate attention as a public health problem. The proportion of non-pregnant women who experience FSD globally, ranges between 40 to 50 percent [2]. The prevalence in Ghana may be higher, varying between 46 to 73 percent [3,4].

In pregnancy, sexual dysfunction(SD) increases remarkably [5] with a reported prevalence of 50–80 percent [6–8]. The prevalence of SD in pregnancy in Egypt is almost 70 percent [8]. In Ghana, the prevalence of SD among pregnant women was unknown until our recent work which reported a prevalence of 65 percent [9]. In spite of this high prevalence, only 12 percent self-reported their SD to their healthcare providers [9] and this could be due to the fact that sexual intercourse in pregnancy and the sexual needs of pregnant women are often not discussed [10]. While normal sexual functioning in pregnancy prepares the couple towards becoming good parents, SD in pregnancy can affect a harmonious relationship between the couple in their preparation for the arrival of their new-born [11]. In addition, unsatisfying sexual life of couples have been associated with marital disharmony, extramarital affairs and broken homes [12].

Socio-demographic factors such as age, educational status, duration of marriage and employment status have all been identified to be associated with the development of FSD [6,13,14]. Pregnancy related factors such as the number of children the woman has, the mode of delivery and the number of previous abortions are also known to have influence on FSD [15]. In addition, socio-cultural factors are known to influence sexual function [16].

Although the prevalence of SD among pregnant women in Ghana is now known [9], the determinants of SD among pregnant women in Ghana is unknown. It is to this end that this study was conducted to identify the factors associated with sexual dysfunction in pregnancy in Ghanaian context. This will enable appropriate measures to be taken to address it.

## Materials and methods

A cross-sectional study using quantitative methods was carried out among pregnant women attending Antenatal Clinic (ANC) of the Greater Accra Regional Hospital (GARH) in 2018. The detailed methods have been reported elsewhere [9]. Briefly, 427 married or cohabiting women who were at least eight weeks pregnant and have been living together with their partners for at least four weeks prior to the survey were consecutively recruited. The Female Sexual Function Index (FSFI), a self-report instrument [17] was used to assess their sexual function.

Pregnant women who had been advised by their healthcare providers to abstain from sexual intercourse, diagnosed with medical conditions, were on medications such as antipsychotics that are known to negatively affect sexual function were excluded.

The study was approved by the Ethics Review Committee of Ghana Health Service (GHS-ERC: 041/12/17) and all the participants provided informed consent.

### Studied variables

The dependent variable of interest was sexual dysfunction. Participants with FSFI score of 26.5 or less were classified as having SD while those with FSFI score greater than 26.5 were classified as not having SD [17].

The independent variables assessed were the respondents’ age and that of their spouses, ethnicity, marital status, duration of marriage or cohabitation and educational status. Others were occupational status, number of deliveries (parity), mode of previous delivery(ies), number of miscarriages, induced abortions, number of lifetime sexual partners, gestational age and body mass index of the respondents.

Pearson’s chi square or Fisher’s exact test, as appropriate was used to tests for association between categorical independent variables and SD. Student’s t-test was used for differences in the means of normally distributed independent continuous variable and the Mann Whitney U Test (Wilcoxon Rank Sum Test) was also used where the assumptions of normality were not met.

Factors which had significant association with SD at the bivariate analysis level and factors which were not significant but have been proven in literature to be associated with sexual dysfunction were all put in a multiple logistic regression model and the adjusted odds ratios and their corresponding 95 percent Confidence Intervals (CI) obtained. The significance of p- value was set at < 0.05 at both the bivariate analysis and multiple logistic regression levels.

## Results

Four hundred and twenty-seven (427) study participants were interviewed. Two of the questionnaires were excluded from the analysis due to incomplete data information, and the remaining 425 were analyzed.

The mean (± SD) age of the respondents was 30.8 (± 4.8) years, (range of 18–45 years). The mean (± SD) age of the spouses of the respondents was 36.4 (± 6.0) years, (range of 22–58 years). The mean (± SD) gestational age of the study participants was 32.3 (±7.1) weeks, (range of 9.7–42.0 weeks). The mean (± SD) BMI of the respondents was 31.1 (± 6.3) kg/m^2^ (range 19.5–53.3 kg/m^2^).

Table 1 shows the details of association between sexual dysfunction and socio-demographic characteristics. At the bivariate analysis level, significant associations were found between sexual dysfunction and the following socio-demographic factors: work status (**χ**^**2**^ = 4.38, p-value = 0.036), marital status (**χ**^**2**^ = 6.25, p-value = 0.012) and duration of stay with partner or spouse (Z score = -2.89, p-value = 0.004). The rest of the socio-demographic factors did not show statistically significant association with SD.

**Table 1:** Association of sexual dysfunction by socio-demographic characteristics of pregnant women attending Antenatal Clinic of Greater Accra Regional Hospital.

Independent Variable	Sexual Dysfunction			
	No n (%)	Yes n (%)	χ^2^	P-value
**Age of respondent (years): Mean** ± SD	30.7 ± 4.7	30.8 ± 4.9	-0.3700^ǂ^	0.7138^§^
**Age of Spouse (years)**	36.4 ± 5.9	36.4 ± 6.1	0.0100^ǂ^	0.9953^§^
**Education**^**¶**^**: Mean** ± SD				
No formal education	5(20.0)	20(80.0)	3.7930	0.1500
≤9 years	59 (33.2)	119 (66.9)		
>9years	85(38.3)	137(61.7)		
**Work status** ^**¶¶**^				
Not working	27(47.4)	30(52.6)	4.3811	**0.0360**
Working	122(33.2)	246(66.9)		
**Ethnicity**				
Akan	84 (41.8)	117 (58.2)	7.9101	0.0950
Ga/Dangme	22(29.7)	52(70.3)		
Ewe	23(27.1)	62(72.9)		
Northern ethnicity	17(31.5)	37(68.5)		
Guan	3(27.3)	8(72.7)		
**Religion**				
Christianity	135 (35.6)	244 (64.4)	0.4844	0.4860
Islam	14 (30.4)	32 (69.6)		
**Marital status**				
Married	125 (38.2)	202(61.8)	6.25	**0.012**
Cohabiting	24 (24.5)	74 (75.5)		
**Duration of stay with partner (years): Median (LQ, UQ)**	3.0(1.0,7.0)	4.8(2.0,8.0)	-2.89^#^	**0.004** ^**¤**^
**Additional number of respondents’ spouses**				
Yes	3 (20.0)	12 (80.0)		0.277^ƪ^
No	146 (35.6)	264 (64.4)		
**Pregnant women’s belief that sex must continue in pregnancy**				
Yes	149 (35.9)	266 (64.1)		0.067^ƪ^
No	0 (0.0)	6 (100.0)		
**Pregnant women who had experienced sexual problem in their pregnancy**				
Yes	8(25.0)	24 (75.0)	1.54	0.215
				
No	141(35.9)	252 (64.1)		

n: Frequency, %: Row percentage, ^¶^Attainment of education up to JHS/Middle school was classified as ≤ 9 years of education and Secondary/vocational school and beyond classified as > 9 years of education, ^¶¶^Not working constituted the unemployed and students, all other forms of occupation were classified as working, § = p-value obtained from Student’s T-Test, ƪ = p-value obtained from Fisher’s exact, ¤ = p-value obtained from Mann Whitney test, ǂ = t- score, # = z- score, SD: Standard deviation, LQ: Lower quartile, UQ: Upper quartile.

n: Frequency, %: Row percentage, ^¶^Attainment of education up to JHS/Middle school was classified as ≤ 9 years of education and Secondary/vocational school and beyond classified as > 9 years of education, ^¶¶^Not working constituted the unemployed and students, all other forms of occupation were classified as working, § = p-value obtained from Student’s T-Test, ƪ = p-value obtained from Fisher’s exact, ¤ = p-value obtained from Mann Whitney test, ǂ = t- score, # = z- score.

Table 2 has the details of association between obstetric /gynaecologic factors and the sexual dysfunction using the Pearson’s Chi square test. There was no statistically significant association between these independent variables and sexual dysfunction.

**Table 2:** Association between sexual dysfunction and obstetric and gynaecologic factors of pregnant women attending Antenatal Clinic of Greater Accra Regional Hospital.

Independent Variable	Sexual Dysfunction in Pregnancy			
	No n (%)	Yes n (%)	χ^2^	P-value
**Gestational age of Index Pregnancy**				
First Trimester	5 (55.6)	4 (44.4)	1.72	0.424
Second Trimester	31 (35.2)	57 (64.8)		
Third Trimester	113 (34.5)	215 (65.6)		
**Parity (Number of deliveries)**				
Nulliparous (No delivery)	45 (37.8)	74 (62.2)	0.62	0.732
Primiparous (One delivery)	47 (34.8)	88 (65.2)		
Multiparous (More than one delivery)	57 (33.3)	114 (66.7)		
**Miscarriages**				
Yes	36 (31.3)	79 (68.7)	0.98	0.323
No	113 (36.5)	197 (63.6)		
**Termination of Unwanted pregnancy**				
Yes	57 (32.8)	117 (67.2)	0.68	0.408
No	92 (36.7)	159 (63.4)		
**Lifetime Sexual Partners**				
One	50 (38.8)	86 (63.2)	1.94	0.585
Two	41 (30.4)	94 (69.6)		
Three	39 (37.9)	64 (62.1)		
More than three	19 (37.3)	32 (62.8)		
**BMI of respondents**				
Normal weight	18 (28.1)	46 (71.9)	2.20	0.333
Overweight	47 (33.8)	92 (66.2)		
Obese	84 (37.8)	138 (62.2)		

n: Frequency, %: Row percentage, **χ**^**2**^: Pearson chi-square value.

Table 3 shows the details of association between the independent variables and SD in pregnancy. Factors which were significantly associated with SD in pregnancy at the bivariate level were tested at multivariate level using logistic regression. Other independent variables which were not statistically significant at the bivariate level but are known to be associated with SD, for example, gestational age [18], age of woman, parity, education [19], body mass index [20], and age of spouse [21] were also included in the model.

**Table 3:** Crude and adjusted odds ratios for factors associated with sexual dysfunction in pregnancy.

Variable	UOR	95% CI	p-value	AOR	95% CI	p-value
**Marital Status**			**0.013** *		-	**0.028** *
Married	1.00			1.00		
Cohabiting	1.91	1.14–3.18		1.88	1.07–3.30	
**Age of respondent**	1.01	0.97–1.05	0.715	1.00	0.93–1.06	0.916
**Age of spouse**	1.00	0.97–1.03	0.995	0.99	0.94–1.04	0.664
**Education** ^**¶**^			0.160			0.397
No formal education	1.00			1.00		
≤9years	0.50	0.18–1.41		0.48	0.16–1.39	
>9years	0.40	0.15–1.11		0.50	0.17–1.46	
**Work status** ^**¶¶**^			**0.038** *		-	0.058
Not working	1.00			1.00		
Working	1.81	1.03–3.19		1.80	0.98–3.30	
**Gestational age**			0.445		-	0.448
First Trimester	1.00			1.00		
Second Trimester	2.30	0.57–9.19		2.28	0.53–9.78	
Third Trimester	2.38	0.63–9.03		2.46	0.60–10.01	
**BMI**			0.335		-	0.256
Normal	1.00			1.00		
Overweight	0.77	0.40–1.46		0.72	0.37–1.42	
Obese	0.64	0.35–1.18		0.59	0.31–1.12	
**Duration of marriage/cohabitation**	1.06	1.01–1.12	**0.019** *	1.08	1.01–1.16	**0.029** *
**Parity**	0.95	0.81–1.12	0.732			0.968
Nulliparous	1.00			1.00		
Primiparous	1.14	0.68–1.90		1.00	0.57–1.75	
Multiparous	1.22	0.75–1.98		0.93	0.48–1.82	

*p-value < 0.05

**p-value<0.01

***p-value<0.001, UOR = Unadjusted Odds Ratio, AOR = Adjusted Odds Ratio, ^¶^Attainment of education up to JHS/Middle school was classified as ≤9 years of education and Secondary/vocational school and beyond classified as > 9 years of education, ^¶¶^Not working constituted the unemployed and students, all other forms of occupation were classified as working.

Both marital status and duration of stay in marriage or cohabitation maintained their significant association with sexual dysfunction in the multiple logistic regression with adjusted odds ratios (AORs) of 1.88 (p-value < 0.050) and 1.08 (p-value < 0.050) respectively. The odds of sexual dysfunction in pregnancy is 88 percent higher among women cohabiting compared with those who were married. Regarding duration of stay with partner, every year of staying together (either in marriage or cohabitation) increases the odds of sexual dysfunction in pregnancy by 8 percent.

No other factor became significant in the multiple logistic regression model.

## Discussion

Socio-demographic factors such as age of the woman and her spouse, marital status, employment status, level of education, religion, ethnicity [22] and duration of marriage [23], have all been reported in literature to be associated with female sexual dysfunction. Gynaecologic and obstetric factors such as parity, previous miscarriages, number of life time sexual partners [15], gestational age [8], and body mass index [20] have also been identified to be associated with sexual dysfunction in pregnancy. However, in this study, only marital status and duration of marriage or cohabitation were significantly associated with sexual dysfunction with following adjusted ORs, 1.88 (CI: 1.07–3.30) and 1.08 (CI:1.01–1.16) respectively.

The odds of sexual dysfunction was 88 percent more among women cohabiting with their partners compared with those who are married. This study included only women who were married or cohabiting and that was used as a proxy for sexual intercourse. Even though some studies have found unclear association between marital status and female sexual dysfunction, systematic review of current literature indicates that marriage is a protective factor against female sexual dysfunction while cohabitation increases the odds of experiencing sexual dysfunction [24] just as was found in the present study.

It has been suggested that marriage provides for the best sexual relationship framework [25]. This could possibly be explained that; cohabiters may suffer from psychosocial uncertainties concerning formalizing their union and that could affect their sexual function. However, further studies would be required to ascertain the definite reasons why cohabiters experience more sexual dysfunction compared to the marrieds.

The duration of staying together (in marriage or cohabitation) remained significant even after controlling for other factors. For every year of stay with a partner or spouse, the odds of SD increases by 8 percent. A positive association between duration of marriage or cohabitation and sexual dysfunction has also been demonstrated in other studies [8,14]. It has been suggested that the reason why sexual dysfunction increases with duration of marriage and cohabitation is due to loss of novelty in sexual life with time [26]. This is sometimes referred to as the loss of ‘‘honeymoon” effect which leads to decrease in satisfaction with sex in the union [26]. It has been found that, at the initial period of marital union, there is a high level of sexual satisfaction because both partners consciously allocate resources and time to their sexual life but with time, this decreases, resulting in reduced frequency of sexual intercourse and sexual satisfaction [25]. Although, the present study did not look at the consequences of sexual dysfunction, Liu has demonstrated that, as marital duration increases, the odds of extramarital affair which has been identified as a major reason for marital dissolution also increases [25]. Therefore, conscious effort and resources should continually be directed to the sexuality of a couple, to maintain better sexual function for as long as possible, even during pregnancy.

The main strength of this study is with the use of a validated a FSFI tool to diagnose SD. Also, to the best of our knowledge, this is the first study that assessed the determinants of SD among pregnant women in Ghana.

The limitations of the study include the failure to consider confounders of FSD such as male sexual dysfunction [27] and female genital mutilation(FGM) [28]. The prevalence of FGM is 1.2% in Accra [29] and therefore we do not think it would have significantly affected the findings. Thirdly, the non-probabilistic selection of the study participants raises the issue of generalizability. However, considering the large sample size, we feel the findings are still relevant. Finally, the study was done in an urban setting and it is not known whether similar findings will be realized in a rural setting with the same tool.

## Conclusion

Cohabiting and increasing length of stay with one’s spouse are significantly associated with SD in pregnancy. Maternal health service providers should bear this in mind when giving care to pregnant women. There is the need for further studies in this population to ascertain the reasons why cohabiting unlike marriage and increasing length of stay with spouse increase a woman’s chance of having SD in pregnancy.

## Supporting information

S1 File Sexual dysfunction in pregnancy questionnaire. (PDF)

S2 File Data set sexual dysfunction in pregnancy. (DTA)

S3 File Published study of prevalence of sexual dysfunction in pregnancy. (PDF)
