# Supplementary material for: Determinants of sexual dysfunction in pregnancy in a large tertiary hospital in Ghana
Source: PLoS One. 2023 Jul 20;18(7):e0288456. doi: 10.1371/journal.pone.0288456 (PMC10358996; doi:10.1371/journal.pone.0288456)
Supplement: S1 File — (PDF) [file pone.0288456.s001.pdf]

PARTICIPANT INFORMATION SHEET AND CONSENT FORM

RESEARCH TOPIC: DETERMINANTS OF SEXUAL DYSFUNCTION IN PREGNANCY AT THE GREATER ACCRA REGIONAL HOSPITAL

INTRODUCTION

I am a student from the University of Ghana, School of Public Health. My telephone number is 0244520182 and email address is mkboamah@gmail.com. I am carrying out a study on the determinants of sexual dysfunction in pregnancy at the Greater Accra Regional Hospital.

NATURE OF RESEARCH

Female sexual dysfunction is defined as a group of problems that affect a woman's desire for sex (libido), sexual arousal, orgasm (i.e. the moment of most intense pleasure in sexual intercourse) and it may or may not involve pain.

Sexual desire or interest is a feeling that includes wanting to have a sexual experience, feeling receptive to a partner's sexual initiation, and thinking or fantasizing about having sex.

Sexual arousal is a feeling that includes both physical and mental aspects of sexual excitement. It may include feelings of warmth or tingling in the genitals, lubrication (wetness), or muscle contractions.

Sexual dysfunction can affect pregnant women as well, but because sex and sexuality appear to be quite private and sensitive, it is hardly discussed while some pregnant women suffer from it silently.

The purpose of the study is to determine the proportion of pregnant women who experience sexual problems, the types of the sexual problems and the associated factors.

STUDY PROCEDURE

This study will involve a total of four hundred and twenty-five (425) respondents who will answer questions about themselves with regard to their sexual activity in pregnancy.
It will take about 30 minutes to complete the interview. It will involve measurement of your weight and height as well answering some questions of female sexuality in pregnancy.
The questions are in two main parts; A and B.

Questions in part A concern socio-demographic characteristics, obstetrics and gynaecology history, culture and sexual activity in pregnancy, religion and sexual activity in pregnancy and your knowledge of sex in pregnancy.

Questions in part B are about female sexual function and the areas which will be explored are desire for sex, sexual arousal, lubrication in sex, orgasm, sexual satisfaction, and pain associated with sexual intercourse.

If there are any questions you would rather not answer or that you do not feel comfortable answering, please say so and we will stop the interview or move on to the next question, whichever you prefer. You will only be required to participate once in this study.

VOLUNTARY PARTICIPATION AND WITHDRAWAL

Your participation in this study is voluntary and you are at liberty not to take part if you wish. You are also assured that, if you decide not to take part, it will not affect the care you will receive in the facility in anyway. Even if you decide to take part, you are also at liberty not to answer any particular question you are uncomfortable with, and you are not required to provide reasons for that. Finally, you are also at liberty to withdraw from the study at any time if you change your mind not to continue with the study and you do not have to provide any reason.

PRIVACY AND CONFIDENTIALITY

The interview will take place in a private room (a consulting room), so that no one else will hear or see you.

You are also assured of confidentiality regarding the information you will provide. Your name will not be taken, however your unique hospital identity number (ID) will be used for identification. This unique ID will be known only to the research team. The questionnaires will be kept in a locked cabinet and access will be restricted to the principal investigator and the supervisor. The data collected will be entered into a personal computer which will be password protected. You are also guaranteed that your name will not appear in any report that will come out from this study and the information gathered will be used solely for the research.

RISKS AND BENEFITS

You will not be exposed to any physical risk if you participate in the study since you will only be answering questions. However, there may be some discomfort in answering some questions about your sexuality. Because of this, female research assistant will conduct the interview to

minimise the risk as much as possible. Again, you will be required to spend about 30 minutes of your time to answer the questions.

Taking part in this study will not give you any direct personal benefit. However, the findings of the study may help address sexual problems of pregnant women.

COMPENSATION

You will not receive monetary payment for participating in the study. However, you will be provided with a bottle of fruit juice and biscuit for your participation.

DECLARATION OF CONFLICT, INTEREST

The researcher declares no conflict of interest and the study is funded entirely by the principal investigator.

This study has been reviewed and approved by Ghana Health Service Ethical Review Committee (GH-ERC) whose tasks are to make sure that research participants are protected from harm and their rights respected.
If you have any questions or concerns, we will be happy to answer or address them now or later. You may contact the administrator, Ghana Health Service Ethical Review Committee, Miss Nana Abena Kwaa Addai-Donkor (0244712919) or Miss Hannah Frimpong (0507041223/0243235225).

PARTICIPANT'S CONSENT FORM

I have read the foregoing information/ the foregoing information has been read to me or translated to me and I have fully understood it.
I consent voluntarily to participate in this study.

Signature/thumbprint (Participant): ____________________________________

Signature/thumbprint (Witness, if translation was done): __________________

Signature of Interviewer: ____

Date: ____

QUESTIONNAIRE ON SEXUAL DYSFUNCTION IN PREGNANCY AT THE GREATER ACCRA REGIONAL HOSPITAL

Questionnaire No………………. **ANC record No…………………**

Weight (Kg)…………… **Height (cm)……………………..**

Gestational age (Weeks)………….LMP……………….1st Trimester scan EDD ………….

PART A

Instructions to the interviewer

1. Circle the response or write in the space provided.

2. Counter check to ensure all the relevant questions are completed.

	**SOCIO-DEMOGRAPHIC CHARACTERISTICS OF RESPONDENTS**			
**NO**	**QUESTION**	**RESPONSE**		
1	How old are you? (Completed years)	……………………………..		
2	What is your highest level of education?	1. No formal education<br>2. Primary	3.Middle/JSS/JHS<br>4. Secondary/Vocational<br>5. Tertiary	
3	What is your religion?	1.Christianity<br>2. Islam	3. Traditional<br>4. Other (Specify)………..	
4	If Christian, what denomination do you belong?	1.Catholic<br>2.Presbyterian<br>3.Methodist<br>4. Anglican	5. Pentecostal<br>6.Charistmatic<br>7. Other (Specify)………<br>………………………….	
5	What is your ethnicity?	1.Akan<br>2.Ga/Dangme<br>3.Ewe	4. Northern ethnicity<br>5.Other (specify)………..<br>………………………….	
6	What is your marital status?	1. Single<br>2. Married<br>3. Cohabiting	4. Separated<br>5. Divorced<br>6. Widowed	

7	How old is your spouse? (completed years)	……………………..		
8	How long have you been married /Cohabiting? (completed years)	……………………….		
9	Does your spouse have additional wife/wives?	1.Yes<br>2.No		
10	If **Yes** to **question 9**, how many more wife/wives does he have?	………………………….		
11	What is your occupation?	1. Unemployed<br>2. Student<br>3. Farmer<br>4. Trader	5. Artisan<br>6. Salaried worker(Public)<br>7.Salaried worker(Private)<br>8. Other (Specify)….<br>……………………	
12	Where do you live?	State ……………………………		
	**OBSTETRIC AND GYAENECOLOGY HISTORY**			
13	How many times have you delivered?	1. None<br>2. One<br>3. Two	4. Three<br>5. Four<br>6. Other (Specify)…….	
	**Skip the next question if you have not delivered before**			
14	What was (were) the mode(s) of delivery(deliveries)?	1.Vaginal birth only<br>2.Caesarean delivery only<br>3.Vaginal and Caesarean delivery		
15	Have you had miscarriage(s) before?	1.Yes<br>2.No		

16	If yes to **question 15**, how many miscarriages have you had?	………………………	
17	Have you terminated pregnancy(ies) you did not want before?	1.Yes<br>2. No	
18	If yes to **question 17,** how many pregnancies have you terminated?	……………………..	
	**CULTURE AND SEXUAL ACTIVITY IN PREGNANCY**		
19.	Does your ethnicity disallow sex in pregnancy?	1.Yes<br>2.No	
20.	If **Yes** to **question 19**, what are the reasons they give?	State reasons (as many as you know)<br>…………………………………………….<br>……………………………………………..<br>…………………………………………………	
	**RELIGION AND SEXUAL ACTIVITY IN PREGNANCY**		
21.	Does your religion disallow sex in pregnancy?	1.Yes<br>2.No	
22	If **Yes** to the **question 21**, what are the reasons they give?	State reasons (as many as you know)<br>……………………………………………<br>……………………………………………<br>…………………………………………….	
	**KNOWLEDGE OF SEX IN PREGNANCY**		
23	What are some of the things you have heard about having sex while pregnant?	State as many as you have heard<br>……………………………………………..<br>……………………………………………..<br>…………………………………………….	

24	In your own view, do you think a woman can continue to have sex during pregnancy?	1.Yes<br>2. No	
25	If Y**es or No** to **question 24,** what are your reasons?	State reasons (as many as possible)<br>………………………………………………<br>………………………………………………	
26	Have you ever had any sexual problem in pregnancy before?	1.Yes<br>2.No	
27	If **Yes** to **question 26,** what was the problem(s)?	……………………………………………………<br>……………………………………………………	
28	If **Yes** to **question 26**, did you report it to any health professional?	1.Yes<br>2.No	
29	If **No**, why?	……………………………………………..	
30	Has a health professional ever discussed sex during pregnancy with you at the antenatal clinic before?	1.Yes<br>2.No	
31	How many sexual partners have you ever had in your life?	………………………..	

PART B- FEMALE SEXUAL FUNCTION INDEX (FSFI)

INSTRUCTIONS: These questions ask about your sexual feelings and responses during the past 4 weeks. In answering these questions, the following definitions apply:

Sexual activity can include caressing, foreplay, masturbation and vaginal intercourse.

Sexual intercourse is defined as penile penetration (entry) of the vagina.

Sexual stimulation includes situations like foreplay with a partner, self-stimulation (masturbation), or sexual fantasy.

Sexual desire or interest is a feeling that includes wanting to have a sexual experience, feeling receptive to a partner's sexual initiation, and thinking or fantasizing about having sex.

Circle one answer per question.

1. Over the past 4 weeks, how often did you feel sexual desire or interest?

    a. Almost always or always
    b. Most times (more than half the time)
    c. Sometimes (about half the time)
    d. A few times (less than half the time)
    e. Almost never or never

2. Over the past 4 weeks, how would you rate your level (degree) of sexual desire or interest?

    a. Very high
    b. High
    c. Moderate
    d. Low
    e. Very low or none at all

Sexual arousal is a feeling that includes both physical and mental aspects of sexual excitement. It may include feelings of warmth or tingling in the genitals, lubrication (wetness), or muscle contractions.

1. Over the past 4 weeks, how often did you feel sexually aroused ("turned on") during sexual activity or intercourse?

a. No sexual activity
b. Almost always or always
c. Most times (more than half the time)
d. Sometimes (about half the time)
e. A few times (less than half the time)
f. Almost never or never

4. Over the past 4 weeks, how would you rate your level of sexual arousal ("turn on") during sexual activity or intercourse?

a. No sexual activity
b. Very high
c. High
d. Moderate
e. Low
f. Very low or none at all

5. Over the past 4 weeks, how confident were you about becoming sexually aroused during sexual activity or intercourse?

a. No sexual activity
b. Very high confidence
c. High confidence
d. Moderate confidence
e. Low confidence
f. Very low or no confidence

6. Over the past 4 weeks, how often have you been satisfied with your arousal (excitement) during sexual activity or intercourse?

a. No sexual activity
b. Almost always or always
c. Most times (more than half the time)
d. Sometimes (about half the time)
e. A few times (less than half the time)
f. Almost never or never

7. Over the past 4 weeks, how often did you become lubricated ("wet") during sexual activity or intercourse?

a. No sexual activity
b. Almost always or always
c. Most times (more than half the time)
d. Sometimes (about half the time)
e. A few times (less than half the time)
f. Almost never or never

8. Over the past 4 weeks, how difficult was it to become lubricated ("wet") during sexual activity or intercourse?

a. No sexual activity
b. Extremely difficult or impossible
c. Very difficult
d. Difficult
e. Slightly difficult
f. Not difficult

9. Over the past 4 weeks, how often did you maintain your lubrication ("wetness") until completion of sexual activity or intercourse?

a. No sexual activity
b. Almost always or always
c. Most times (more than half the time)
d. Sometimes (about half the time)
e. A few times (less than half the time)
f. Almost never or never

10. Over the past 4 weeks, how difficult was it to maintain your lubrication ("wetness") until completion of sexual activity or intercourse?

a. No sexual activity
b. Extremely difficult or impossible
c. Very difficult
d. Difficult
e. Slightly difficult
f. Not difficult

11. Over the past 4 weeks, when you had sexual stimulation or intercourse, how often did you reach orgasm (climax)?

a. No sexual activity
b. Almost always or always
c. Most times (more than half the time)
d. Sometimes (about half the time)
e. A few times (less than half the time)
f. Almost never or never

12. Over the past 4 weeks, when you had sexual stimulation or intercourse, how difficult was it for you to reach orgasm (climax)?

a. No sexual activity
b. Extremely difficult or impossible
c. Very difficult
d. Difficult
e. Slightly difficult
f. Not difficult

13. Over the past 4 weeks, how satisfied were you with your ability to reach orgasm (climax) during sexual activity or intercourse?

a. No sexual activity
b. Very satisfied
c. Moderately satisfied
d. About equally satisfied and dissatisfied
e. Moderately dissatisfied
f. Very dissatisfied

14. Over the past 4 weeks, how satisfied have you been with the amount of emotional closeness during sexual activity between you and your partner?

a. No sexual activity
b. Very satisfied
c. Moderately satisfied
d. About equally satisfied and dissatisfied
e. Moderately dissatisfied
f. Very dissatisfied

15. Over the past 4 weeks, how satisfied have you been with your sexual relationship with your partner?

a. Very satisfied
b. Moderately satisfied
c. About equally satisfied and dissatisfied
d. Moderately dissatisfied
e. Very dissatisfied

16. Over the past 4 weeks, how satisfied have you been with your overall sexual life?

a. Very satisfied
b. Moderately satisfied
c. About equally satisfied and dissatisfied
d. Moderately dissatisfied
e. Very dissatisfied

17. Over the past 4 weeks, how often did you experience discomfort or pain during vaginal penetration?

a. Did not attempt intercourse
b. Almost always or always
c. Most times (more than half the time)
d. Sometimes (about half the time)
e. A few times (less than half the time)
f. Almost never or never

18. Over the past 4 weeks, how often did you experience discomfort or pain following vaginal penetration?

a. Did not attempt intercourse
b. Almost always or always
c. Most times (more than half the time)
d. Sometimes (about half the time)
e. A few times (less than half the time)
f. Almost never or never

19.Over the past 4 weeks, how would you rate your level (degree) of discomfort or pain during or following vaginal penetration?

a. Did not attempt intercourse
b. Very high
c. High
d. Moderate
e. Low
f. Very low or none at all

Thank you for completing this questionnaire
