# Supplementary material for: Determinants of sexual dysfunction in pregnancy in a large tertiary hospital in Ghana
Source: PLoS One. 2023 Jul 20;18(7):e0288456. doi: 10.1371/journal.pone.0288456 (PMC10358996; doi:10.1371/journal.pone.0288456)
Supplement: S3 File — (PDF) [file pone.0288456.s003.pdf]

Received: 25 January 2020 | Revised: 24 May 2020 | Accepted: 15 July 2020
DOI: 10.1002/ijgo.13314

CLINICAL ARTICLE
Obstetrics

WILEY International Journal of GYNECOLOGY OBSTETRICS FIGO

Prevalence of sexual dysfunction among prenatal women attending the Greater Accra Regional Hospital, Ghana

Ernest T. Maya[1] | Martin O. Boamah[2,*] | Kofi Agyabeng[1] | Emmanuel Srofenyoh[2] | Kareem Mumuni[3] | Ali Samba[3]

[1]School of Public Health, University of Ghana, Accra, Ghana
[2]Department of Obstetrics and Gynecology, Greater Accra Regional Hospital, Accra, Ghana
[3]Department of Obstetrics and Gynecology, University of Ghana Medical School, Accra, Ghana

*Correspondence
Martin O. Boamah, Reproductive Health and Family Planning Unit, Department of Obstetrics and Gynecology, Greater Accra Regional Hospital, Accra, Ghana.
Email: mkboamah@gmail.com

Abstract

Objective: To estimate the prevalence and types of sexual dysfunction in pregnancy.

Methods: A cross-sectional facility-based descriptive study among pregnant women attending the prenatal clinic of the Greater Accra Regional Hospital, a large tertiary health facility in Accra, Ghana, from May to June 2018. The inclusion criteria were 18 years or older, singleton pregnancy of 8 gestational weeks or more, and residing with their partner for at least 4 weeks before the study. Face-to-face interviews were conducted among consecutively enrolled women by using the Female Sexual Function Index (FSFI) tool.

Results: Overall, 425 women were enrolled. The mean age was 30.8 ± 4.8 years. The mean gestational age was 32.3 ± 7.1 weeks (range 9.7–42.0 weeks). The prevalence of sexual dysfunction in pregnancy was 64.9% (95% confidence interval [CI], 60.3%–69.4%) but only 32 (7.5%) women self-reported sexual problems. The predominant types of sexual disorder were desire disorder (377 [88.7%; 95% CI, 85.3%–91.4%] women) and arousal disorder (320 [75.3%; 95% CI, 71.0%–79.2%]).

Conclusion: Sexual dysfunction in pregnancy was found to be common, but most pregnant women were not aware that they had it.

KEYWORDS
Female Sexual Function Index; FSFI; Pregnancy; Prevalence; Sexual disorders; Sexual dysfunction

1 | INTRODUCTION

Sexual dysfunction generally refers to a group of disorders that affect sexual desire (libido), arousal, and orgasm, and that may or may not involve pain.[1] Although the condition affects both sexes, females experience it more.[2,3]

Female sexual dysfunction (FSD) can be regarded as an umbrella term that encompasses problems associated with sexual desire (hypoactive sexual desire disorder), arousal (female sexual arousal disorder), orgasm (female orgasmic disorder), and pain disorders (dyspareunia).[4] These conditions are not mutually exclusive and a woman may have multiple disorders at the same time.[5]

Although more attention is being paid to the importance of FSD,[6] sexual dysfunction in pregnancy remains largely uninvestigated. Pregnancy itself has been found to worsen sexual function for women, owing to the physiologic, physical, and psychologic changes that take place.[7] Sexual activity and intercourse are considered safe in pregnancy and do not predispose a woman to adverse pregnancy outcomes if she has no pre-existing complications.[7]

Although FSD has been investigated among non-pregnant women in Ghana, it is not known how FSD affects pregnant women. Knowing the prevalence and types of sexual dysfunction among pregnant women will help providers to give appropriate care. The aim of the present study was therefore to estimate the prevalence

 © 2020 International Federation of Gynecology and Obstetrics

and types of sexual dysfunction in pregnancy in a population in Ghana.

2 | MATERIALS AND METHODS

2.1 | Study design and population

The present cross-sectional descriptive study was carried out from May 14 to June 25, 2018, among pregnant women receiving routine prenatal care at the Greater Accra Regional Hospital, Accra, Ghana, a tertiary hospital that provides prenatal care for approximately 14 000 pregnant women annually. Ethical approval to conduct the study was obtained from the Ethical Review Committee of Ghana Health Service, Accra (GHS-ERC: 041/12/17). Written consent was obtained from all participants, who were informed that participation in the research was voluntary, and that whether they participated or not would have no bearing on the services that they received at the health facility.

Women were eligible if they were at least 18 years old, were carrying a single fetus with a gestational age of 8 weeks or more, and had been living with their spouse or partner for at least 4 weeks before the study. Living together was used as a proxy for sexual activity and a minimum of 4 weeks of staying together was chosen because the Female Sexual Function Index (FSFI) tool used assesses sexual activity in the preceding 4 weeks.[8] Pregnant women who had been asked by their healthcare providers to abstain from sexual intercourse at any point within the 4 weeks, those with medical conditions diagnosed within or before the 4 weeks prior to the study, and those on antipsychotic drugs, antidepressants, or any medication that adversely affects sexual function were excluded from the study.

2.2 | Sample size

A minimum sample size of 384 was calculated based on a prevalence of 0.50 in the population, an absolute precision error of 0.05, and a 95% confidence level. The minimum sample size was adjusted by 11% to allow for non-responders and incomplete questionnaires to determine a target sample size of 427.

2.3 | Enrollment and data collection

Study participants were recruited from women attending the prenatal clinic. Two midwives from the Korle-Bu Teaching Hospital in Accra, who were trained as research assistants, reviewed the women's maternal health records to identify potentially eligible participants and described the study to them. Women who showed interest were invited individually into a dedicated study room, where they were taken through a comprehensive informed consent process. Those who consented were interviewed individually by using the FSFI questionnaire and data were collected on socio-demographic characteristics, sexual desire, arousal, lubrication, orgasm, sexual satisfaction, and sexual pain among other factors. Participants were enrolled consecutively due to the short time (6 weeks) for data collection and the large sample size required.

The weight and height of the participants were measured and their body mass index (BMI, calculated as weight in kilograms divided by the square of height in meters) was determined. Gestational age was calculated by using the date of the participant's last menstrual period if she was certain about it, or by the first-trimester ultrasound scan otherwise. The questions were in English but, for participants who did not understand English, they were translated into the local languages (Twi, Ga, and Ewe) by the research assistants who were fluent in English and these local languages.

2.4 | Study variables

Sexual dysfunction was assessed by using the six domains of the FSFI tool (desire, arousal, lubrication, orgasm, sexual satisfaction, and sexual pain). Responses to each question were scored on a Likert-scale from 0 (no sexual activity) or 1 (suggestive of dysfunction) to 5 (suggestive of normal sexual function). Each domain's score was obtained by adding the scores of the individual questions in that domain and multiplying the sum by a domain factor provided in the FSFI scoring chart.[8] Scores less than 65% of the maximum achievable score of 6 in each domain (i.e., 3.9) were categorized as sexual disorder in that domain; all other scores were categorized as no disorder.[9]

The overall FSFI score for each participant was obtained by adding the scores of the six domains to obtain a total that theoretically ranged

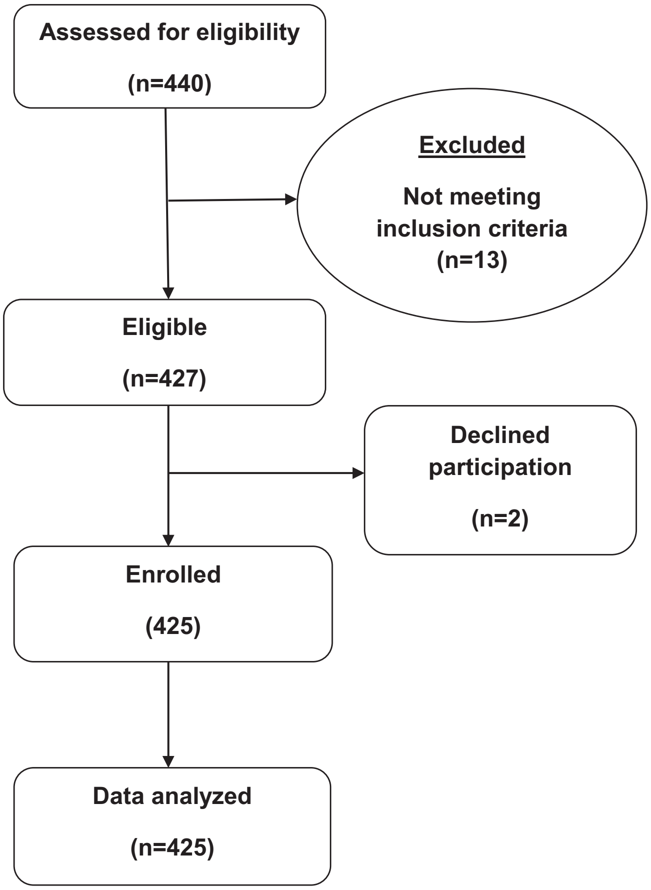

FIGURE 1 Flowchart showing recruitment of participants.

TABLE 1 Sociodemographic characteristics of the study women.[a]

Characteristic	Value (n=425)
Age, y	30.8 ± 4.8
<20	2 (0.5)
20–24	31 (7.3)
25–29	142 (33.4)
30–34	160 (37.7)
35–39	73 (17.2)
40–44	16 (3.8)
>44	1 (0.2)
Partner age, y	36.4 ± 6.0
20–24	5 (1.2)
25–29	43 (10.1)
30–34	115 (27.1)
35–39	136 (32.0)
40–44	81 (19.1)
45–49	34 (8.0)
50–54	5 (1.2)
55–59	6 (1.4)
Educational status	
No formal education	25 (5.9)
Primary	29 (6.8)
Middle/JSS/JHS	149 (35.1)
Secondary	103 (24.2)
Tertiary	119 (28.0)
Occupation	
Unemployed	41 (9.7)
Student	16 (3.8)
Trader	134 (31.5)
Artisan	114 (26.8)
Salaried worker	111 (26.1)
Other[b]	9 (2.1)
Ethnicity	
Akan	201 (47.3)
Ga/Dangme	74 (17.4)
Ewe	85 (20.0)
Guan	11 (2.6)
Northern ethnicity	54 (12.7)
Religion	
Christianity	379 (89.2)
Islam	46 (10.8)
Marital status	
Married	327 (76.9)
Cohabiting	98 (23.1)
Gestational age, weeks	
8–13 (1st trimester)[c]	9 (2.1)
14–27 (2nd trimester)	88 (20.7)
≥28 (3rd trimester)	328 (77.2)
BMI	31.1 ± 6.3
18.6–25.0 (normal weight)	65 (15.3)
25.1–30.0 (overweight)	139 (32.7)
≥30.1 (obese)	221 (52.0)

(Continues)

TABLE 1 (Continued)

Abbreviations: BMI, body mass index (calculated as weight in kilograms divided by the square of height in meters); JHS, Junior High School; JSS, Junior Secondary School.
[a]Values are given as mean ± SD or number (percentage).
[b]Farmer, head porter.
[c]The 1st trimester was considered to be 8–13 weeks because only women with a gestational age of at least 8 weeks were enrolled.

from 2 to 36 points. A total FSFI score of 26.5 or less was considered to be diagnostic of FSD.[10]

2.5 | Data analysis

The completed questionnaires were coded and the data were entered into Excel (Microsoft, Redmond, WA, USA) on a password-protected personal computer. The data were imported into STATA version 15 (StataCorp, College Station, TX, USA) for cleaning and analysis.

TABLE 2 FSFI domain scores, types of sexual disorder, and prevalence of sexual dysfunction among the study women.[a]

Variable	Value (n=425)
Domain score	
Desire	3.2 ± 0.9 (1.2–6.0)
Arousal	3.2 ± 1.0 (0.6–6.0)
Lubrication	4.0 ± 1.2 (0.0–6.0)
Orgasm	4.2 ± 1.2 (0.0–6.0)
Sexual satisfaction	5.0 ± 0.9 (0.8–6.0)
Sexual pain	5.1 ± 1.3 (0.0–6.0)
FSFI score	24.7 ± 4.2 (6.0–34.8)
Types of sexual disorder[b]	
Desire disorder (score <3.9)	377 (88.7; 85.3–91.4)
Arousal disorder (score <3.9)	320 (75.3; 71.0–79.2)
Lubrication disorder (score <3.9)	155 (36.5; 31.9–41.2)
Orgasmic disorder (score <3.9)	121 (28.5; 24.4–33.0)
Sexual pain (score <3.9)	86 (20.2; 16.7–24.3)
Sexual dissatisfaction (score <3.9)	52 (12.2; 9.3–15.7)
Overall prevalence of FSD	
Sexual dysfunction (FSFI ≤26.5)	276 (64.9; 60.26–69.4)
Normal sexual function (FSFI >26.5)	149 (35.1; 30.65–39.7)

Abbreviation: FSD, female sexual dysfunction; FSFI, Female Sexual Function Index.
[a]Values are given as mean ± SD (range) or number (percentage; 95% confidence interval).
[b]A participant may have multiple sexual disorders; the percentage of each type of sexual disorder was assessed for all 425 participants.

Descriptive statistics, including number (percentage), mean ± SD, and confidence intervals (CIs) were used to summarize the data. Cronbach α values were calculated to assess the reliability of the FSFI tool among pregnant women; an α value of 0.70 and above was considered to be acceptable.[11]

3 | RESULTS

During the study period, 427 pregnant women were recruited; two women subsequently refused consent and the data for 425 were analyzed. Participant recruitment and data analysis are shown in Figure 1. The mean ± SD age of the participants was 30.8 ± 4.8 years (range 18–45 years); the gestational age was 32.3 ± 7.1 weeks (range 9.7–42.0 weeks); and the BMI was 31.1 ± 6.3 (range 19.5–53.3) (Table 1).

The FSFI domain scores, types of sexual disorder, and prevalence of FSD are summarized in Table 2. The sexual disorders identified were desire, arousal, orgasm, and sexual pain disorders. The sexual domains that had the lowest mean scores were desire and arousal (3.2 ± 0.9); the domain with the highest score was sexual pain (5.1 ± 1.2).The most common sexual disorder was desire disorder, reported among 377 (88.7%; 95% CI, 85.3%–91.4%) women; by contrast, the least common was sexual pain disorder, reported among 86 (20.2%; 95% CI, 16.7%–24.3%) women. Overall, 155 (36.5%; 95% CI, 32.0%–41.2%) respondents experienced vaginal lubrication difficulties, and 52 (12.2%; 95% CI, 9.4%–15.7%) were dissatisfied with their overall sex life. Among those who experienced sexual desire disorder, the highest numbers were in the extreme age groups (<20 and >44 years) (Fig. 2). In total, 30 (7.1%) women experienced all four recognized sexual disorders, and 28 (6.6%) women did not have a sexual disorder.

Overall, the mean FSFI score among the study participants was 24.7 ± 4.2. The prevalence of sexual dysfunction using a cutoff point of 26.5 was 64.9% (95% CI, 60.3%–69.4%; n=276 women). The prevalence of sexual dysfunction was highest among those who were at least 40 years (Fig.2). Figure 3 shows that, with the exception of lubrication difficulties, sexual disorders and dysfunction were more prevalent among pregnant women in their second (14–27 weeks) and third (≥28 weeks) trimesters. With regard to body size, the highest prevalence of FSD (47/65, 72.3%) was found among women with normal BMI (Fig. 4).

The reliability score of the FSFI tool in this study setting was good (α>0.70). The Cronbach α values obtained for each domain of sexual

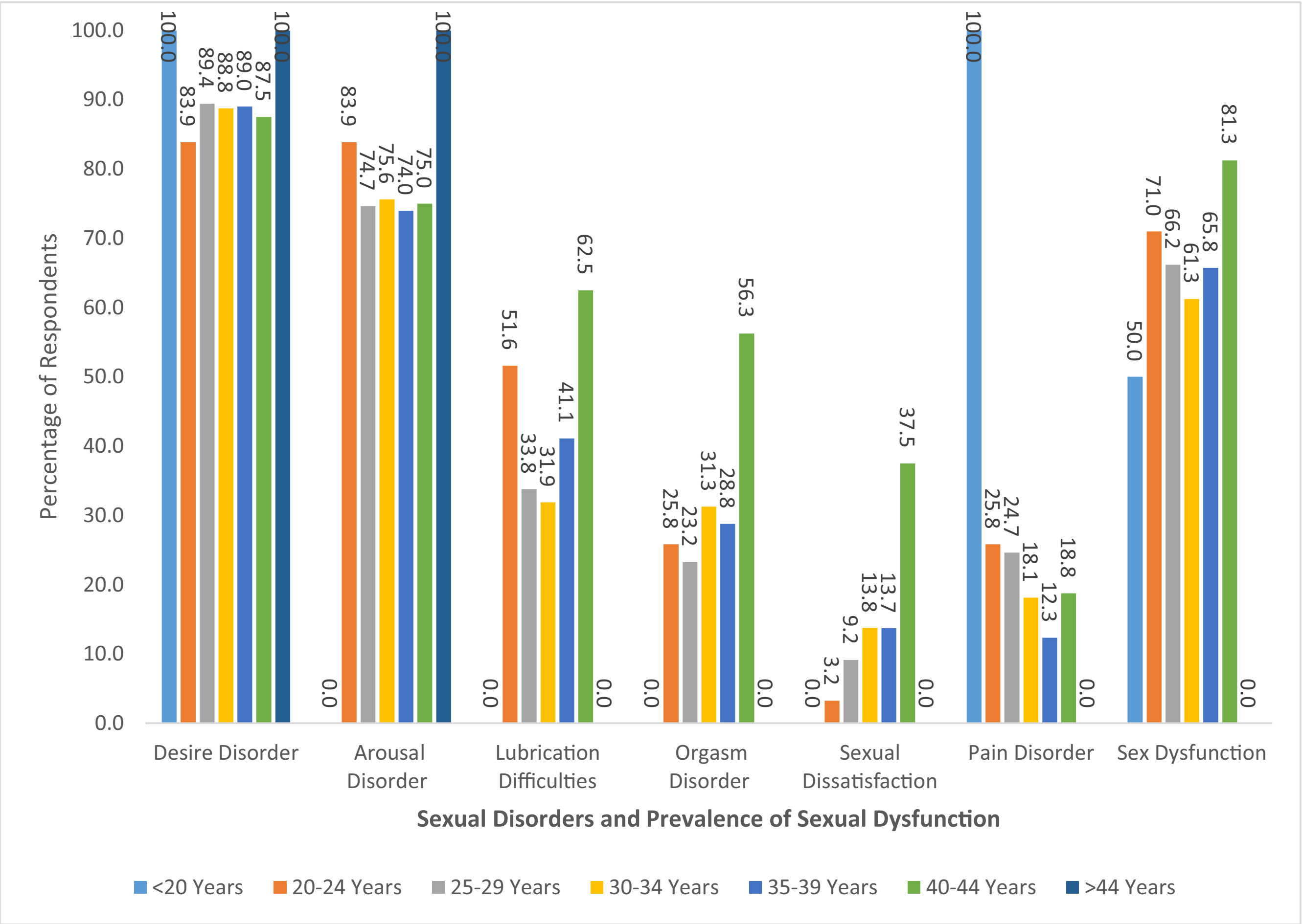

FIGURE 2 Prevalence of sexual disorders and dysfunction by age among pregnant women attending the prenatal clinic of Greater Accra Regional Hospital.

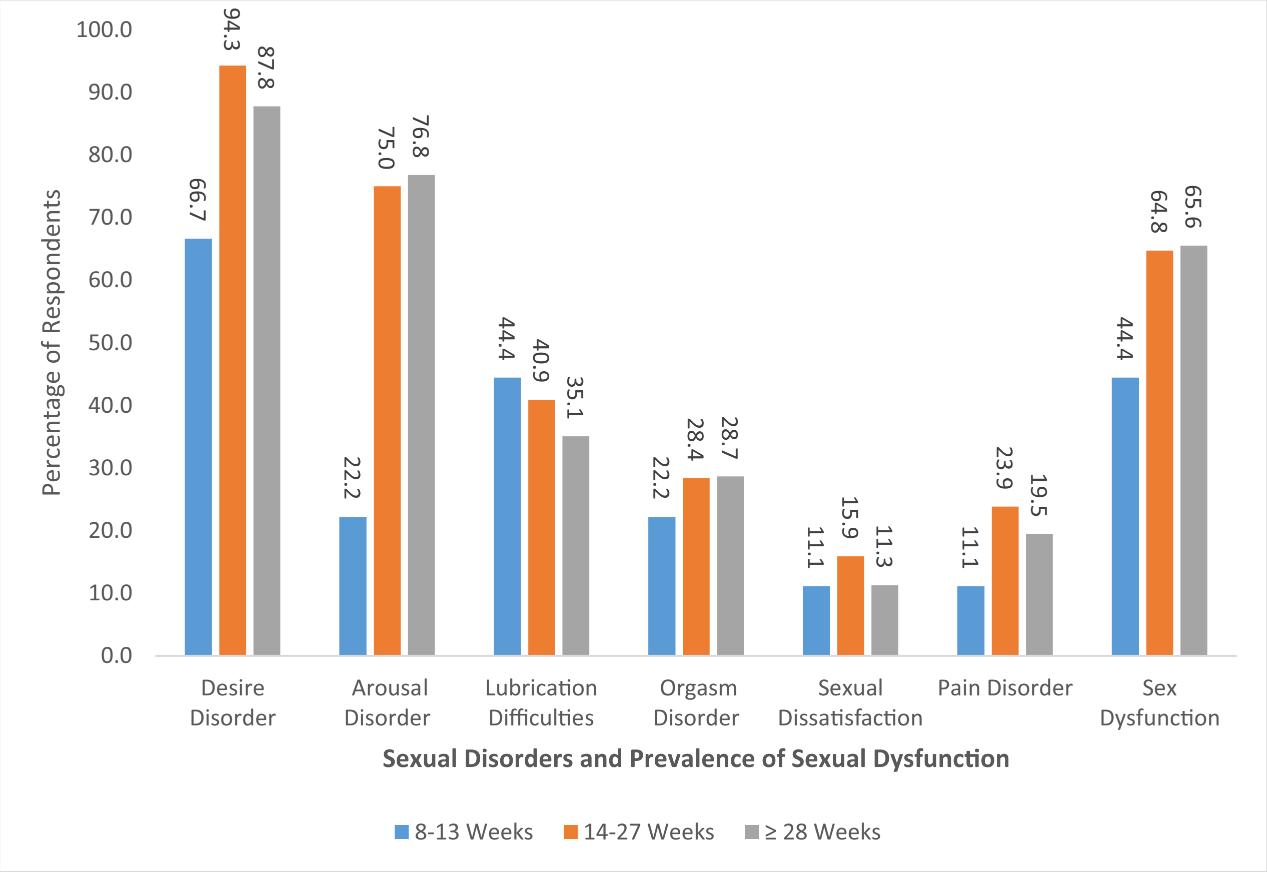

FIGURE 3 Prevalence of sexual disorders and dysfunction by gestational age among pregnant women attending the prenatal clinic of Greater Accra Regional Hospital.

disorder and overall sexual dysfunction ranged from 0.84 to 0.96 (Table 3), indicating that the FSFI tool was consistent in measuring sexual dysfunction in the study setting.

More than half of the participants (266/425, 62.6%) reported that health professionals at the prenatal clinic briefly discussed sexual activity in pregnancy with them. Among the 425 participants, 32 (7.5%) self-reported sexual problems that they had experienced in the current pregnancy (Table 4). The most common type of sexual problem self-reported by the 32 respondents was painful sex (26/32, 81.3%). More than half of the women (21/32, 65.6%) did not report the problem to a health professional. The most frequently cited reason for not reporting sexual problems to health professionals was that the women thought it was normal to experience such problems in pregnancy (10/21, 47.6%).

4 | DISCUSSSION

The present study found that the prevalence of sexual dysfunction in pregnancy among women as determined by the FSFI was 64.9% (95% CI, 60.3%–69.4%), but only 32 (7.5%) women self-reported sexual problems. The predominant types of sexual disorder were desire disorder (377, 88.7%; 95% CI, 85.3%–91.4%) and arousal disorder (320, 75.3%; 95% CI, 71.0%–79.2%).

The lower mean scores of sexual desire and arousal disorders found in the study agree with a Turkish study, which reported that the mean scores for all sexual domains, and particularly desire and arousal, were much lower among pregnant than among non-pregnant women.[12] Furthermore, the present finding that sexual disorders were more prevalent among women in their second and third trimesters agrees with data from a meta-analysis on sexual function and pregnancy.[13] The reason why sexual disorders, particularly desire and arousal disorders, are more common in pregnancy and even higher in the second and third trimesters

TABLE 3 Reliability test of FSFI domains.

Domain	Cronbach α
Desire	0.89
Arousal	0.84
Lubrication	0.90
Orgasm	0.84
Satisfaction	0.92
Pain	0.96
Overall	0.89

Abbreviation: FSFI, Female Sexual Function Index.

TABLE 4 Self-reported sexual problems in pregnancy and reasons for not reporting among the study women.

Variable	Total no. of women	No. (%)
Types of sexual problem reported[a]	32	
Lower abdominal pain and discomfort during and or after sex		26 (81.3)
Vaginal bleeding during or after sex		3 (9.4)
Extreme fatigue during and after sex		3 (9.4)
Low libido		2 (6.3)
Inability to reach orgasm		1 (3.1)
Lubrication difficulties		1 (3.1)
Reasons for not reporting self-recognized sexual problems to health professional	21	
Thought it was normal		10 (47.6)
No apparent reason		5 (23.8)
The problem resolved spontaneously		3 (14.3)
Treated the problem herself		2 (9.5)
Felt embarrassed to talk to a doctor about it		1 (4.8)

[a]Participants could report multiple responses.

might be due to the presence of discomfort, fatigue, and changes in self-image in pregnancy that worsen as gestational age advances.[13]

Although at least three-quarters of the participants experienced sexual desire and or arousal disorder, less than one-quarter experienced sexual pain or orgasm disorder. This may suggest that, although a significant proportion of pregnant women do not desire sex or get sexually aroused, once they are engaged in sexual activity, they do not experience pain and they go on to achieve orgasm. It is

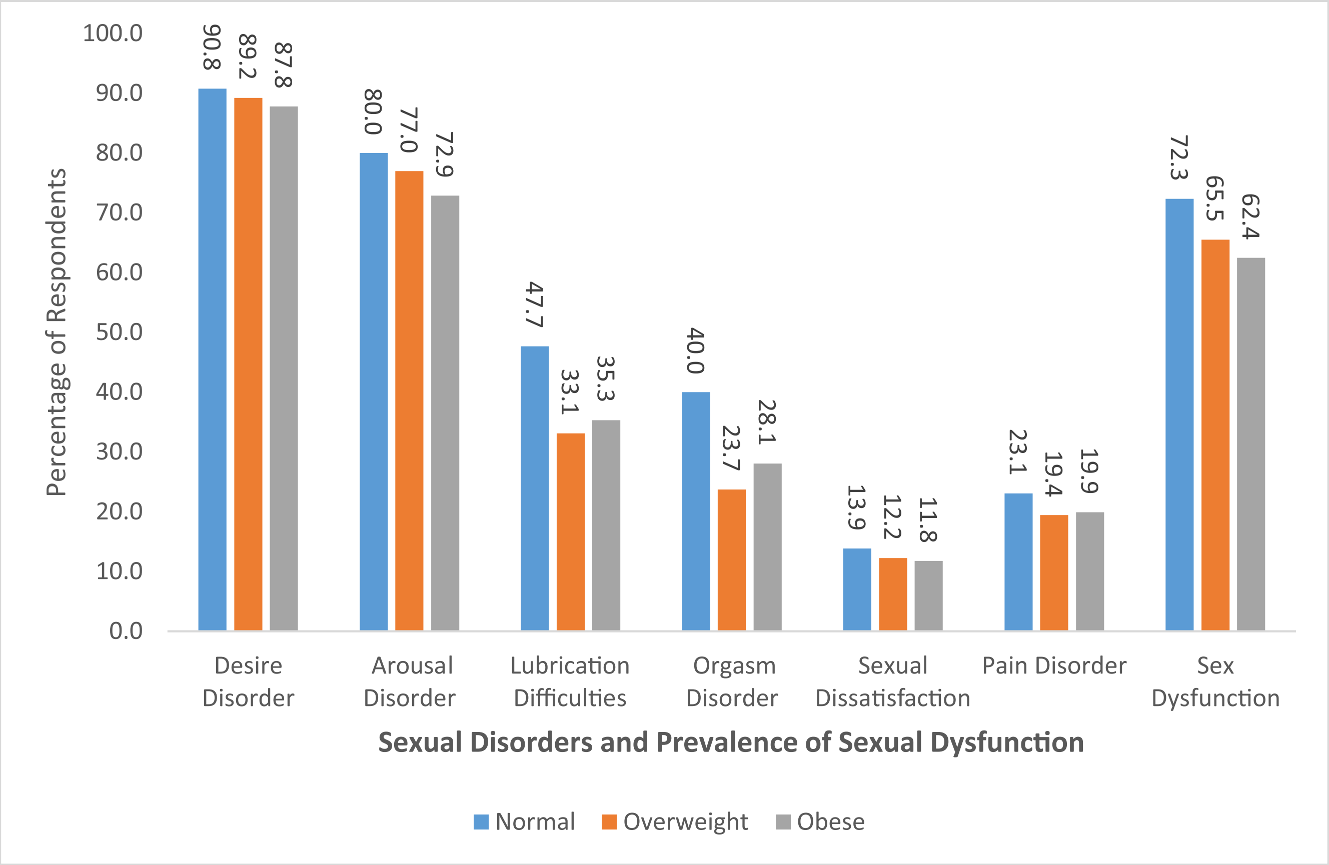

FIGURE 4 Prevalence of sexual disorders and dysfunction by BMI among pregnant women attending the prenatal clinic of Greater Accra Regional Hospital. Abbreviation: BMI, body mass index (calculated as weight in kilograms divided by the square of height in meters).

therefore not unexpected that approximately 90% of study women were satisfied with their overall sexual life in pregnancy.

The prevalence of sexual dysfunction of 65% found in the study is similar to the level of 69% reported among pregnant women in Egypt,[14] but is much higher than the value of 53% reported among non-pregnant women in Nigeria.[15] In Ghana, Afrane et al.,[16] also using the FSFI tool, recorded a prevalence of 46% among non-pregnant women. The present study therefore supports the assertion that sexual dysfunction is higher among pregnant women than non-pregnant women in Ghana.

Possible reasons why sexual dysfunction is more prevalent among pregnant women than among their non-pregnant counterparts include unfounded concerns regarding adverse obstetric events, such as fear of pregnancy loss during sexual intercourse in pregnancy.[13] Heaviness from maternal weight gain has also been suggested to make sexual activity in pregnancy uncomfortable and to impair sexual function.[14]

In the present study, FSD was more common among pregnant women with normal BMI (47/65, 72.3%) than among those who were either overweight or obese. This finding agrees with other studies that did not demonstrate a higher prevalence of FSD in overweight and obese non-pregnant women in France[17] and Egypt.[18] By contrast, Naldoni et al.[19] reported that FSD was more common among pregnant women with excessive weight gain. The present study evaluated absolute BMI rather than weight gain, which might explain the difference observed.

A few studies have reported a prevalence of sexual dysfunction among non-pregnant women that is higher than 60%.[2,20,21] For example, in two different studies among Ghanaian non-pregnant women, Amidu et al.[2,21] reported a prevalence of 69%– 73%. The higher prevalence reported by Amidu et al. might be due to their use of a different diagnostic instrument (Golombok Rust Inventory of Sexual Satisfaction, GRISS), which measures other domains of sexual function. Whereas FSFI measures desire, arousal, lubrication, orgasm, satisfaction, and pain, GRISS measures anorgasmia, vaginismus, sexual frequency, sexual communication, satisfaction, non-sensuality, and sexual avoidance. In addition, GRISS is used to evaluate a couple, whereas FSFI is used to assess women only,[22] which might account for the differences in findings.

Despite the large proportion of pregnant women experiencing at least one sexual disorder in the present study, only 7.5% of them self-reported their sexual problems. More than half of those who had sexual problems did not report them to a health professional, either for no apparent reason or because they thought that it was normal in pregnancy. This might be due to a lack of detailed education on sexual activity during pregnancy because sexual activity in pregnancy is not included in the topics that are listed in Ghana's maternal health records for discussion during prenatal visits.

The strengths of the study include the use of a standardized FSFI tool with validated cutoff points for diagnosis of sexual dysfunction and domain disorders,[10] unlike many other sexuality measurement instruments. Indeed, the Cronbach α values obtained for each domain of sexual disorder and overall sexual dysfunction (range 0.84–0.96) reflect the high internal consistency of the FSFI tool in diagnosing sexual dysfunction in the study population.

The study also has limitations. First, male sexual dysfunction, which is a possible trigger for FSD,[23] was not considered. Second, female genital mutilation, which can cause FSD,[24] was not considered; however, this is unlikely to affect the findings significantly because the prevalence of female genital mutilation in Accra is only 1.2%.[25] Third, the participants were not randomly selected, raising the issue of study generalization; however, the findings are supported by the large sample size. Last, the study women were from urban settings and it is not known whether the tool will perform similarly in a rural setting.

5 | CONCLUSION

The study found that, among pregnant women in Ghana, sexual dysfunction in pregnancy is common but many women are unaware that they have it. The most common types of sexual disorder in pregnancy were desire and arousal disorders, and women in the second and third trimesters were the most affected.

Pregnant women should be educated on sexual function in pregnancy and should be encouraged to report their sexual problems to healthcare providers. It will be important to conduct further studies using the FSFI tool in a randomized sample and also in different settings. Lastly, future studies must look at the factors associated with sexual dysfunction and disorders among pregnant women to enable appropriate counselling and management.

AUTHOR CONTRIBUTIONS

MOB and ETM conceived and designed the study with contributions from ES, KM, and AS. MOB and ETM conducted the study. KA analyzed the data. MOB wrote the manuscript. ETM, ES, KM, and AS revised the manuscript. All authors read and approved the final manuscript.

ACKNOWLEDGEMENTS

The authors thank Mary Asare and Priscilla Dansu for assistance in the data collection process; and the staff of the Greater Accra Regional Hospital Antenatal Clinic, which is headed by Amina Yakubu, for cooperation and help during data collection.

CONFLICTS OF INTEREST

The authors have no conflicts of interest.

REFERENCES

1. Bhugra D, Colombini G. Sexual dysfunction: Classification and assessment. *Adv Psychiatr Treat*. 2013;19:48–55.
2. Amidu N, Owiredu WKBA, Woode E, et al. Incidence of sexual dysfunction: A prospective survey in Ghanaian females. *Reprod Biol Endocrinol*. 2010;8:106.

3. Amidu N, Owiredu W, Addai-Mensah O, Gyasi-Sarpong K, Alhassan A. Prevalence of male sexual dysfunction among Ghanaian populace: Myth or reality? *Int J Impot Res*. 2010;22:337–342.
4. Pronier C, Monk-Turner E. Factors shaping women's sexual satisfaction: A comparison of medical and social models. *J Gend Stud*. 2014;23:69–80.
5. Stern TA, Cassem NH, Rosenbaum JF, Fricchione GL, Jellinek MS, eds. *Massachusetts General Hospital Handbook of General Hospital Psychiatry*, 6th edn. Massachusetts: Saunders; 2010:323–335.
6. McCool ME, Zuelke A, Theurich MA, Knuettel H, Ricci C, Apfelbacher C. Prevalence of female sexual dysfunction among premenopausal women: A systematic review and meta-analysis of observational studies. *Sex Med Rev*. 2016;4:197–212.
7. Zakšek TŠ. Sexual activity during pregnancy in childbirth and after childbirth. *Sexol Midwifery*. 2016;6:111–133.
8. Rosen R, Brown C, Heiman J, et al. The Female Sexual Function Index (FSFI): A multidimensional self-report instrument for the assessment of female sexual function. *J Sex Marital Ther*. 2000;26:51–65.
9. Tehrani FR, Farahmand M, Simbar M, Afzali HM. Factors associated with sexual dysfunction; A population based study in Iranian reproductive age women. *Arch Iran Med*. 2014;17:3–9.
10. Wiegel M, Meston C, Rosen R. The Female Sexual Function Index (FSFI): Cross-validation and development of clinical cutoff scores. *J Sex Marital Ther*. 2005;31:1–20.
11. Tavakol M, Dennick R. Making sense of Cronbach's alpha. *Int J Med Educ*. 2011;2:53–55.
12. Aydin M, Cayonu N, Kadihasanoglu M, Irkilata L, Atilla MK, Kendirci M. Comparison of sexual functions in pregnant and non-pregnant women. *Urol J*. 2015;12:2339–2344.
13. Yeniel AÖ, Petri E. Pregnancy, childbirth, and sexual function: perceptions and facts. *Int Urogynecol J*. 2013;25:5–14.
14. Ahmed M, Madny E, Ahmed WAS. Prevalence of female sexual dysfunction during pregnancy among Egyptian women. *J Obstet Gynaecol Res*. 2014;40:1023–1029.
15. Nwagha UI, Oguanuo TC, Ekwuazi K, Olubobokun TO, Nwagha TU, Onyebuchi AK. Prevalence of sexual dysfunction among females in a university community in Enugu. *Nigeria. Niger J Clin Pract*. 2014;17:791–796.
16. Afrane BA, Kretchy IA, Imbeah EP, et al. Prevalence and self-management of female sexual dysfunction among women in six regions of Ghana: A cross- sectional study. *World J Pharm Res*. 2016;5:241–254.
17. Bajos N, Wellings K, Laborde C, Moreau C. Sexuality and obesity, a gender perspective: Results from French national random probability survey of sexual behaviours. *Br Med J*. 2010;340:2573.
18. Mostafa AM, Khamis Y, Helmy HK, Arafa AE, Abbas AM. Prevalence and patterns of female sexual dysfunction among overweight and obese premenopausal women in Upper Egypt; a cross sectional study. *Middle East Fertil Soc J*. 2018;23:68–71.
19. Naldoni LMV, Pazmiño MAV, Pezzan PAO, Pereira SB, Duarte G, Ferreira CHJ. Evaluation of sexual function in Brazilian pregnant women. *J Sex Marital Ther*. 2011;37:116–129.
20. Wolpe RE, Zomkowski K, Silva FP, Queiroz APA, Sperandio FF. Prevalence of female sexual dysfunction in Brazil: A systematic review. *Eur J Obstet Gynecol Reprod Biol*. 2017;211:26–32.
21. Amidu N, Owiredu WK, Gyasi-Sarpong CK, Woode E, Quaye L. Sexual dysfunction among married couples living in Kumasi metropolis. *Ghana. BMC Urol*. 2011;11:3.
22. Meston CM, Derogatis LR. Validated instruments for assessing female sexual function. *J Sex Marital Ther*. 2002;28(S1):155–164.
23. Aslan E, Nezihe KB, Gungor I, Kadioglu A, Birsen KD. Prevalence and risk factors for low sexual function in women: A study of 1,009 women in an outpatient clinic of a university hospital in Istanbul. *J Sex Med*. 2008;5:2044–2052.
24. Ismail SA, Abbas AM, Habib D, Morsy H, Saleh MA, Bahloul M. Effect of female genital mutilation/cutting; Types I and II on sexual function: Case-controlled study. *Reprod Health*. 2017;14:1–6.
25. Ghana Statistical Service. *Ghana Multiple Indicator Cluster Survey with an Enhanced Malaria Module and Biomarker, 2011*. Accra, Ghana: Final Report; 2011.
